# Knowledge and transmission risk awareness of tuberculosis among the pilgrims attending a religious mass gathering in India: a cross-sectional study

**DOI:** 10.1186/s12889-021-12192-8

**Published:** 2021-11-22

**Authors:** Tim Bäckdahl, Megha Sharma

**Affiliations:** 1 Department of Global Public Health- Health Systems and Policy (HSP): Medicines, focusing antibiotics, Karolinska Institutet, Stockholm, Sweden; 2 Department of Pharmacology, R. D. Gardi Medical College, Ujjain, Madhya Pradesh India

**Keywords:** Tuberculosis, Mass gathering, *Kumbh Mela*, Knowledge of tuberculosis, Prevention of infection, Ujjain, India

## Abstract

**Background:**

Tuberculosis infection accounts for more annual deaths than any other single infectious disease, except the Corona Virus infection. It is a significant global issue and India is one of the most affected countries. Religious mass gatherings congregate millions of pilgrims at one place. Over-crowding is a high-risk factor for the transmission of tuberculosis. Knowledge and awareness of the disease are proven prerequisites for the spread-prevention and early diagnosis of tuberculosis. The present study was designed to explore the knowledge of tuberculosis and awareness of disease transmission risk among pilgrims attending a religious mass gathering, the *Kumbh Mela* (2016) in Ujjain, India.

**Methods:**

Self-reported data on the pilgrims’ tuberculosis-knowledge were collected using a cross-sectional study design. A contextual, pre-tested questionnaire was used, using the convenience sampling method. In addition to the anonymous descriptive analyses, a composite knowledge-score was developed to enable comparisons between demographic groups.

**Results:**

In total, 1665 pilgrims participated in the study with 38.8 years of mean age and 59% literacy rate. The most recognized symptoms of tuberculosis were “Cough for more than 15 days with sputum” (94%) and “Blood in sputum” (81%). Most participants knew that tuberculosis is an infectious disease (93%) and not hereditary (91%). Additionally, 84% considered it is a potentially lethal disease that requires extensive treatment. However, vaccine awareness was poor (4%). “Direct contact with tuberculosis patient” (78%) and “To be in a crowded area” (4%) were considered as the most important risk factors for tuberculosis transmission. For the composite knowledge-score, a significantly higher mean score was seen among those with at least ten years of schooling compared with illiterates (*p* <  0.001). Occupation and residency also affected the mean score.

**Conclusion:**

Most pilgrims attending the *Kumbh Mela* had basic knowledge of the characteristics of tuberculosis, but some important knowledge gaps concerning the transmission risks associated with crowded situations existed. These gaps need to be addressed in future policies to enable safer mass gatherings and to end the TB epidemic, globally.

## Background

The World Health Organization’s (WHO) End TB Strategy and the Sustainable Development Goal (target 3.3) are operational to limit the incidences of tuberculosis (TB) by 2030 [1, 2]. Even though the infection rates are slowly decreasing worldwide, TB is still accounted for more annual deaths than any other single infectious disease, except for Coronavirus disease (COVID-19) recently [2]. Low- and middle-income countries are most affected by TB and India is the most affected country in the world [2, 3].

Revised National TB Control Program (RNTCP), and National Strategic Plan for Tuberculosis Elimination (NSP for *TB* elimination *2017–2025)* were launched by the Indian government with a vision to end TB in the country by 2025 [4]. The programs focus on awareness, early detection, and treatment compliance to achieve the goals [1, 5]. Studies from various settings show that lack of knowledge, insufficient awareness, and widespread stigma surrounding TB are the main causes for misinterpretation of the severity of the situation [5–12]. Thereby, delay in diagnosis and poor adherence to treatment regimens leads to increased risk for the spread of infection, poorer outcomes, and a high burden of the disease.

Large crowds i.e. mass gatherings (MG) are correlated with risks for public health that includes the risk of communicable diseases, dehydration, intoxication, violence, injuries and so on [13]. In 2010, a Lancet conference adopted the *Jeddah declaration* establishing MG medicine as a field of research [14]. Concerns have been raised in recent years especially regarding the knowledge and potential globalized transmission of infectious diseases, both endemic and emerging [15]. The current COVID-19 pandemic highlights the risk even further [16]. Studies state that the special circumstances of a religious MG, with stress, physical exhaustion and poor nutrition may compromise the immune response of people with latent TB causing reactivation of the bacteria [17]. This is further theorized to increase the risk of TB-spread during these events [17]. The majority of the previous studies investigated the spread of TB during the *Hajj* pilgrimage in Saudi Arabia and found that pilgrims had acquired latent TB 3 months after the  pilgrimage [18]. Thus, there is an absolute need for more research regarding the knowledge and attitude of TB to understand the magnitude of the problem.

The studies conducted in one of the MG in India (*Kumbh Mela*) showed cholera outbreaks as the most commonly associated public health issue, due to poor access to clean water and sanitation, and stampede incidents [19–22]. However, the research focusing on TB among the pilgrims at *Kumbh Mela* is scarce [23].

### Aim

To estimate the knowledge of TB and awareness of the risk of disease transmission, among pilgrims at a mass gathering event, the *Kumbh Mela,* in Ujjain, India.

## Methods

### Study design and data collection tool

This study was designed as a cross-sectional questionnaire-based study to generate descriptive data on the self-reported knowledge and awareness around TB. The target population was limited to pilgrims attending the 2016 *Kumbh Mela* in Ujjain, India.

A questionnaire was designed to achieve the aim of the study. The questionnaire includes universal TB-knowledge related questions that have been used in previous studies [9, 10]. The questionnaire was pre-tested among the local population in two rounds, to check the comprehensibility and validity (data not included in the present study). The questionnaire was modified based on the pilot-test results to provide maximum information. The final questionnaire contained 35 questions. A mix of closed (Yes/No), multiple-choice and open-ended questions were included in the questionnaire. Answers for the open-ended questions were coded and categorized, to enable the quantitative analysis. The questionnaire was printed in *Hindi* to facilitate better understanding by the participants and was later translated into English for data entering and analyses purposes.

The questions were formulated to assess the participants’ knowledge about symptoms, etiology, and severity of TB (9 questions), treatment of TB (6 questions), methods of transmission and participants’ personal experiences and attitudes towards TB (6 questions). Binary variables were generated for closed (Yes/No) questions, while multiple-choice answers were entered as nominal categorical variables. In addition, basic demographic data were also collected such as the age, sex, place of residence, participant’s education, occupation, number of family members and annual income of the family.

### Study setting

#### Kumbh Mela

The *Kumbh Mela* is a month-long Hindu pilgrimage held every twelfth year in each of the four holy Indian cities; Nashik, Haridwar, Prayagraj and Ujjain. It is considered the largest religious congregation (MG) on Earth and has been deemed an Intangible Cultural Heritage by UNESCO [24]. In 2016, tens of millions of people gathered in Ujjain, to perform one of the main rituals i.e. to bathe in the holy *Kshipra* river [25]. Unlike the *Hajj*, the pilgrims attending the *Kumbh Mela* are mostly travelling from within the country without the need for special permits, making surveillance and organization of visitors more challenging [26].

#### Sampling population

A non-probability sampling method, based on the convenience approach, was used to recruit the participants. As per the scale and short timeframe of the festival, it was the only practically feasible method for this study. All attempts were made to get most of the participants recruited in the study during the period.

#### Data collection

The data was collected by four trained persons. Four places nearby to the riverbanks were selected for the foreseen reasons (more pilgrims passing by for bathing) to collect the data in the *Kumbh Mela* area *(2016)*. The data collectors were given rotational shift work at selected places to overcome the probability of selection bias. The passing-by pilgrims were approached randomly. After explaining the aim of the study, they were asked for their willingness to participate in the study. Those who voluntarily consented were included in the study. Literate participants were given the questionnaire and a pen to respond on their own, while the illiterate participants received assistance for reading the questions and recording their responses. Participants were encouraged but not forced, to respond to all questions.

#### Analysis

Since this was a cross-sectional study with a single group (pilgrims attending the *Kumbh Mela*), the focus was on descriptive statistics. Some of the multiple-choice questions are presented as bar graphs for an easier overview. The Chi^2^-test was used to calculate statistical differences in frequency between males and females for all questions.

Additionally, a composite knowledge-score was calculated from the responses provided for the knowledge-based questions. This variable was created during the secondary analysis of the data to enable comparison of the knowledge between different demographic groups. Twenty-seven knowledge-based questions were selected to compare the knowledge between different demographic groups. Therefore, the maximum possible knowledge-score, if all questions would have been answered correctly, was 27. All these questions were selected to get a broad depiction of the participant’s knowledge of the topic. The selected questions contained both binary and multiple-choice questions. Each correct answer, as indicated by the responder was given a score of one while all the other responses were given a score of zero. For example, the question “For how long should the standard TB treatment be taken?” was a multiple-choice question and the correct response, 6–8 months, generated one point while the other alternatives were given a score of zero. A few questions had more than one correct answer. Therefore, all correct answers were given a score of one. For example, the question ‘Symptoms of TB infection’ had several correct answers such as cough for more than 15 days, recurrent fever, unintentional weight loss, pain in the chest, difficulty in breathing, and weakness, thus each correct response was given a score of one.

The Student’s t-test or the one-way ANOVA with the Bonferroni correction for pairwise comparison was used to test for statistical significances. The results were compared within the demographic groups.

#### Ethical approval

Ethical approval for this study was acquired from the Institutional Ethics Committee (IEC) of R.D. Gardi Medical College (RDGMC) in Ujjain, India. IEC reference number 58/2016.

All methods were carried out in accordance with the relevant guidelines and regulations. As per recommendations of the R.D. Gardi Medical College (RDGMC) Ethics committee, informed written consent was obtained from all literate participants and informed oral consent was obtained from the illiterate participants prior to data collection. Participants were not offered any economic benefit or reimbursement to participate in the study. Participants were ensured for the maintenance of full confidentiality of the data. The data was anonymized by giving a code number to each participant which was used while performing the analyses. Moreover, the data were analyzed in groups to minimize the risk of breach of confidentiality.

## Results

### Demographics

In total, 1665 pilgrims willingly participated in the study. Their ages ranged from 15 to 99 years, with a mean of 38.8 years. Males were more prevalent than females in the sample (58 and 42% respectively). Education level varied, but the majority (75%) had less than ten years of schooling and 41% were illiterate. The most common occupation was self-employment, and most of the participants were from the Ujjain district or other districts of Madhya Pradesh (Table 1).

**Table 1:** Demographic characteristics of the study participants attending a mass gathering, *Kumbh Mela*, in Ujjain, India

Variable	Description of variable	n (%)
Sex	Male	960 (58%)
(*N* = 1665)	Female	705 (42%)
Age	Mean	38.8 years
(*N* = 1664)	Standard deviation (SD)	15.1 years
	Median	36 years
	Range	15–99 years
	Youth (15–24)	292 (18%)
	Adult (25–64)	1257 (75%)
	Elderly (65+)	115 (7%)
Education	Illiterate^*^	636 (41%)
(*N* = 1539)	Primary (1st -5th grade)	211 (14%)
	Secondary (6th – 9th grade)	301 (20%)
	Upper secondary (10th – 12th grade)	296 (19%)
	Graduation	69 (4%)
	Post-graduation	26 (2%)
Occupation	Government job	28 (2%)
(*N* = 1515)	Private sector job	474 (31%)
	Self-employed	549 (36%)
	Business	54 (4%)
	Housewife	335 (22%)
	Student	75 (5%)
Residence	Ujjain city	340 (21%)
(*N* = 1665)	Ujjain district	547 (33%)
	Other districts in Madhya Pradesh	720 (43%)
	Other states in India	49 (3%)

^*^Illiteracy is defined as reporting no formal education and not being able to independently read and answer the questionnaire

### Knowledge questions

#### Symptoms

**Fig. 1:**
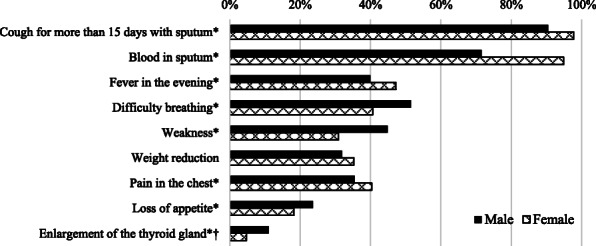
The proportion of participants responding to the question- ‘What are the symptoms of TB?’ and recognizing the symptoms associated with TB, by sex. *Statistically significant difference between the sexes (*p* <  0.05 using Chi^2^-test). *N* = 1665 participants. †Enlargement of the thyroid gland is a rare symptom of TB. Several options can be selected by the participants

The most recognized symptoms of TB were “cough for more than 15 days with sputum” and “blood in sputum” (94 and 81% respectively). The long-term effects of the disease, such as “loss of appetite” and “weight reduction” were less well known (21 and 33% respectively). Symptoms like “blood in sputum” were markedly more recognized by females than males, while “weakness” was more frequently reported by males (Fig. 1).

#### Treatment

In general, the awareness about the availability of vaccines for TB was low (4%). Most pilgrims seem aware of the requirements of long treatment to tackle TB (6–8 months, 66%) and that it is not acceptable to skip medication during the treatment period (97%). A slim majority of the participants stated that TB-treatment is not pricey for the patients (54%). Most of the participants (84%) stated that they would suggest a TB-patient to get the treatment at a government hospital rather than a private facility. Twelve percent of participants believed that bathing in the river during the *Kumbh Mela* can cure a disease like TB (Table 2).

**Table 2:** Knowledge about the etiology, treatment and severity of tuberculosis among the participants attending a mass gathering (*Kumbh Mela*) in Ujjain, India

Questions related to-	Response	Males n (%)	Females n (%)	Total n (%)	*P*-value*
*Treatment of TB*					
- Is there a vaccine for TB?	Yes	54 (5)	8 (1)	62 (4)	< 0.001
	No	669 (70)	442 (63)	1111 (67)	
	Don’t know	237 (25)	255 (36)	492 (29)	
- For how long the standard TB treatment should be taken?	1 month	6 (1)	3 (0)	9 (1)	< 0.001
	2–3 months	171 (18)	12 (2)	183 (11)	
	6–8 months	597 (62)	507 (72)	1104 (66)	
	1 year	167 (17)	177 (25)	344 (21)	
	Don’t know	19 (2)	6 (1)	25 (1)	
- Is it acceptable to skip treatment for a day or two?	Yes	40 (4)	15 (2)	55 (3)	0.021
	No	920 (96)	690 (98)	1610 (97)	
- Is the treatment for TB costly to the patient?	Yes	61 (6)	26 (4)	87 (5)	0.007
	No	491 (51)	405 (57)	896 (54)	
	Don’t know	408 (43)	274 (39)	682 (41)	
- Where would you suggest someone, go and get TB treatment?	Government hospital	807 (84)	590 (84)	1397 (84)	0.837
	Private hospital	153 (16)	115 (16)	268 (16)	
- Can a disease like TB be cured by bathing in a holy river?	Yes	99 (10)	108 (15)	207 (12)	< 0.001
	No	809 (84)	590 (84)	1399 (84)	
	Don’t know	52 (5)	7 (1)	59 (4)	
*Etiology and severity of TB*					
- Is TB an infectious disease?	Yes	890 (93)	655 (93)	1545 (93)	0.795
	No	70 (7)	49 (7)	119 (7)	
- Is TB a hereditary disease?	Yes	91 (9)	58 (8)	149 (9)	0.376
	No	869 (91)	647 (92)	1516 (91)	
- Is TB a preventable disease?	Yes	902 (94)	688 (98)	1590 (95)	< 0.001
	No	36 (4)	6 (1)	42 (3)	
	Don’t know	22 (2)	11 (2)	33 (2)	
- What kind of disease is TB? *(Several options can be selected)*	Uncomplicated	54 (6)	1 (0)	55 (3)	< 0.001
	Lethal	769 (80)	624 (89)	1393 (84)	
	Curable but long treatment	730 (76)	661(94)	1391 (84)	
- Is TB a life-threatening disease?	Yes	725 (75)	537 (76)	1262 (76)	0.760
	No	235 (25)	168 (24)	403 (24)	
- Is it possible to live a normal life with TB?	Yes	590 (62)	403 (57)	993 (60)	< 0.001
	No	282 (29)	293 (42)	575 (34)	
	Don’t know	88 (9)	9 (1)	97 (6)	
- What proportion of the total Indian population has a TB infection?	10%	197 (21)	180 (26)	377 (22)	0.029
	20%	195 (20)	116 (16)	311 (19)	
	40%	21 (2)	10 (1)	31 (2)	
	Don’t know	547 (57)	399 (57)	946 (57)	
- A TB patient is vulnerable to other diseases such as …?	AIDS	164 (17)	155 (22)	319 (19)	< 0.001
	High blood pressure	153 (16)	16 (2)	169 (10)	
	Jaundice	110 (12)	66 (9)	176 (11)	
	Other	7 (1)	0 (0)	7 (0)	
	Don’t know	526 (55)	468 (66)	994 (60)	
- According to you, which disease is more dangerous: TB or cancer?	TB	111 (12)	163 (23)	274 (16)	< 0.001
	Cancer	837 (87)	542 (77)	1379 (83)	
	Don’t know	12 (1)	0 (0)	12 (1)	

*P-values for the difference between sexes (using Chi^2^-test). *N* = 1665 participants, *AIDS* Acquired immunodeficiency syndrome, *TB* tuberculosis

#### Etiology and severity

Most participants described TB as infectious disease (93%) but not hereditary (91%). The majority of the participants (95%) thought TB is a preventable disease whereas 84% of participants considered TB as a potentially lethal disease that requires extensive and long treatment. However, almost a quarter of the participants (24%) indicated that it is not a life-threatening infection. Regarding the prospect to live a normal life with TB infection, 60% responded that it is possible.

Only 2% of pilgrims knew the answer for the proportion of the total Indian population with a TB infection, while 41% underestimated the prevalence [27]. Most participants (60%) were unsure of the vulnerability of the TB patients for other diseases. When given the choice between cancer and TB, 83% considered cancer as the more dangerous disease (Table 2).

#### Transmission

**Fig. 2:**
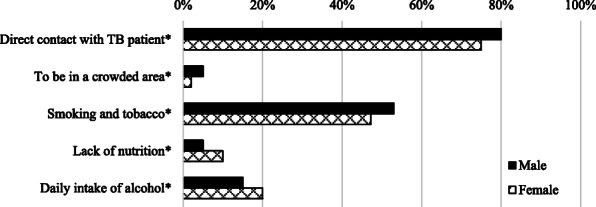
The sex-wise proportion of participants’ response for the most important **risk factors** for the transmission of tuberculosis*.* *Statistically significant difference between sex (*p* <  0.05 using Chi^2^-test). *N* = 1665 participants, several options could be selected by the participants, TB = tuberculosis

Figure 2 presents the pilgrim’s awareness of TB transmission. Of the alternatives given, 78% answered “direct contact with TB patient” to be the most important reason for the spread of TB. Half of the respondents answered that “smoking and tobacco” was a key reason for TB infection.

**Fig. 3:**
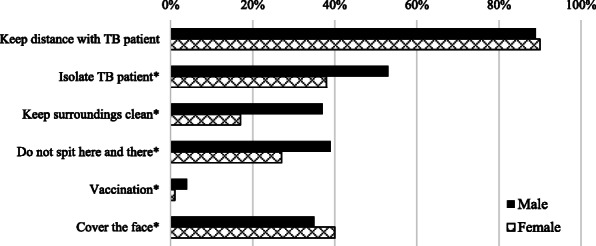
The sex-wise proportion of participants’ responses for the general measures useful to avoid the spread of tuberculosis*.* *Statistically significant difference between sex (*p* <  0.05 using Chi^2^-test). *N* = 1665 participants. Several options could be selected by the participants, TB = tuberculosis

Figure 3 presents the general precautionary measures to be used to avoid getting TB infection. The most frequently selected answer (89%) was “keeping distance with TB patient”. More males than females indicated that “clean surroundings” and “not spitting here and there” are important measures to avoid spreading TB (*p* <  0.05). Once again, vaccination was not recognized as a measure to prevent TB by most of the participants.

### Attitudes and experiences

More males than females (52 vs 36% respectively) said they will not feel comfortable staying with a TB patient. In addition, 4% of the participants (72/1624) listed themselves as TB patients and 201 participants stated that they know a TB patient. Overall, 8% of the participants (132/1659) reported that they know a person who has had TB before who is now TB-free. Regarding the possibility to get infected if bathing in the river together with a TB patient, 1% thought there is a risk of getting the infection. All pilgrims participating in the study had planned to bathe in the holy river (Table 3).

**Table 3:** Personal attitudes and experiences of the participants regarding tuberculosis in a mass gathering in Ujjain, India

Question	Response	Males n (%)	Females n (%)	Total n (%)	*P*-value*
- Are you comfortable staying with a person with TB?	Yes	497 (52)	257 (36)	754 (44)	< 0.001
	No	109 (11)	109 (16)	218 (13)	
	Don’t know	354 (37)	339 (48)	693 (42)	
- Do you know any TB patients? (*n* = 1624)	Yes	132 (14)	69 (10)	201 (12)	0.007
	No	791 (82)	632 (89)	1423 (86)	
- Are you a TB patient? (*n* = 1657)	Yes	56 (6)	16 (2)	72 (4)	< 0.001
	No	898 (94)	687 (97)	1585 (95)	
- Do you know anyone who had TB before, but is now “TB-free”? (*n* = 1659)	Yes	33 (3)	1 (0)	34 (2)	NA
	Yes, myself	4 (0)	12 (2)	50 (3)	
	Yes, family member	35 (4)	13 (2)	48 (3)	
	No	848 (88)	679 (96)	1527 (82)	
- If a TB patient takes a bath in the river with you, do think there is a possibility you could get infected?	Yes	13 (1)	6 (1)	19 (1)	0.618
	No	285 (30)	214 (30)	499 (30)	
	Don’t know	662 (69)	485 (69)	1147 (69)	
- Do you plan to take a bath in the holy river?	Yes	959 (100)	704 (100)	1663 (100)	0.826
	No	1 (0)	1 (0)	2 (0)	

*P-values for the difference between sexes using Chi^2^-test. NA = statistical comparison between sexes not performed. Unless otherwise stated, all 1665 participants gave answers to each of these questions, *TB* tuberculosis

### Knowledge-score

**Fig. 4:**
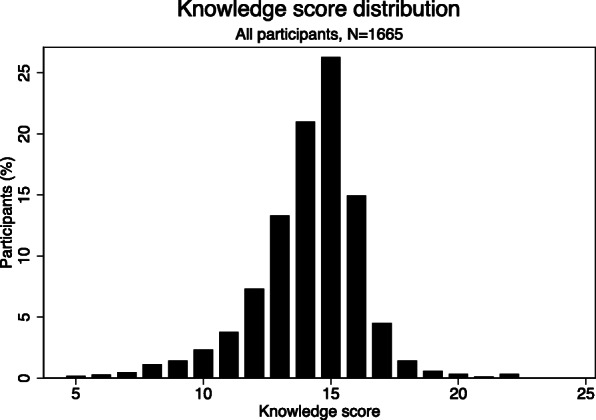
Distribution of knowledge scores among all participants regarding tuberculosis in a mass gathering in Ujjain, India

The composite knowledge-score was normally distributed among the participants with a mean score of 14.1 (14.0–14.2, 95% confidence interval, CI) and a standard deviation of +/− 2.1 (Fig. 4 and Table 4). The highest knowledge-score was 22.0 and the lowest was 5.0. The interquartile range (Q1-Q3) was 13.0 to 15.0.

**Table 4:** Overall knowledge score of the participants, with measures of central tendency and dispersion for all participants, followed by demographic categories sorted by mean score

All participants		Score	
	Mean (SD)	14.1 (2.1)	
	Confidence interval (95%)	14.0–14.2	
	Median	14.0	
	Range	5.0–22.0	
	Interquartile range (Q1-Q3)	13.0–15.0	
Demographic groups		Score, mean (SD)	*p*-value*
Sex	Male	14.1 (2.4)	
	Female	14.2 (1.6)	0.535
Age	Elderly (65+)	13.9 (1.8)	
	Adults (25–64)	14.1 (2.2)	0.843
	Youths [15–24]	14.2 (2.1)	0.631
Education	Illiterate	13.9 (2.0)	
	Primary (1st -5th grade)	14.0 (1.9)	1.000
	Secondary (6th – 9th grade)	14.1 (2.2)	1.000
	Upper secondary (10th – 12th grade)	14.5 (2.3)	0.001**
	Graduation	15.2 (2.5)	< 0.001**
	Post-graduation	15.2 (2.7)	0.057
Occupation	Self-employed	13.8 (2.1)	
	Business	14.0 (2.6)	1.000
	Housewife	14.3 (1.6)	0.006**
	Private sector job	14.4 (2.1)	< 0.001**
	Government job	15.0 (2.6)	0.062
	Student	15.0 (2.0)	< 0.001**
Residence	Other states in India	12.8 (3.4)	
	Other districts in Madhya Pradesh	14.0 (2.2)	0.001**
	Ujjain district	14.3 (1.9)	< 0.001**
	Ujjain city	14.5 (1.9)	< 0.001**

*For the sex-variable Student’s t-test was used to test for significance. For the other variables, *P*-values show if there was a significant difference in mean score compared with the lowest scoring category within each group (using one-way ANOVA with the Bonferroni correction for pairwise comparison). **Considered significant, *p* < 0.05. SD = standard deviation, Q = quartile. The maximum score was 27.0

There was no significant difference in the overall knowledge-score between males and females, nor among the different age groups. On the other hand, the level of education did make a difference. Pilgrims who had completed upper secondary- or graduation level of education scored significantly higher (on average 1.3 points) than those with no formal education (illiterates). In addition, the respondents’ self-reported occupation was associated with a difference in the scores. The self-employed participants had the lowest average (13.8 points) and scored significantly lower than housewives and those with private-sector jobs. The students had the highest significant score (15.0 points on average). Residency affected, with those coming from the states in India other than Madhya Pradesh scoring significantly lower than those living nearby to the study site (Table 4). The response rates on most of the open-ended questions such as household income, number of family members, were low (< 0.5%) therefore, analysis of the economic impact on knowledge-score could not be made.

## Discussion

To the best of our knowledge, this is the first study that provides an insight into the knowledge, risk awareness and attitudes regarding TB among the participants attending one of the world’s largest MG. Though there are some widely shared understandings about TB in this population, important knowledge gaps still exist and need to be addressed in future policy to enable safe MG, prevent the spread of infection and achieve the goal of ending the TB epidemic in India.

### Knowledge on symptoms and treatment of TB

The majority of pilgrims appear to know that TB is an infectious but preventable disease and that the most widely recognized symptom is a productive cough lasting more than two weeks. These findings could suggest that the information campaign by RNTCP, focusing especially on symptom recognition, have successfully reached out with this message [28]. Additionally, most participants in this study seem to be aware that TB is a potentially life-threatening disease that spreads through direct contact with infected individuals and that keeping a distance from TB patients is a way to avoid transmission.

Compared with the findings from Huddart et al. (2013–14), the participants in our study reported higher levels of knowledge in key areas (≥80%, Figs. 1 and 2). In the previous study, only 53% recognized cough as a symptom of TB and no more than 67% knew it is a communicable disease [9]. In a household survey conducted by Sreeramareddy et al. (2005–06), only 56% reported that TB spreads between people through coughing in the air [29]. This difference could, in part reflect on improved population-level knowledge on TB since that time, with reservations for the differences in methodology of data collection, which complicates the comparison.

Other important symptoms such as fever and unintended weight reduction were less recognized by the participants in the present study. This could be due to less emphasis on these symptoms in the government’s campaigns. Interestingly, blood in the sputum was reported as a symptom by significantly more females than males, suggesting a gender bias in how well this symptom was noticed, which is not present as a common symptom in TB patients. Perhaps this was remembered due to the dramatic way of presenting blooded cough as a signal of TB infection in Television-shows and movies [30]. This highlights the influence of media to shape people’s attitudes, behaviors, and knowledge of diseases and suggest emphasizing an advantageous approach for future information campaigns.

Concerning the treatment of TB, most pilgrims seem aware that it takes over 6 months of medication to treat and cure the disease. Compared with Huddart et al., fewer people knew about the correct period of treatment in this study, although Huddart et al. interviewed actual TB-patients just starting their treatment [9]. The Indian government offers free diagnostics and treatment to the registered TB-patients, moreover, they get a small amount of money from the government to support the dietary requirements. Yet, it is worth noticing that only half of the respondents knew that the treatment for TB is not costly. There is a need to promote this fact since it could be a potential barrier to seek care among the poorer, and vulnerable population who want to avoid catastrophic spending on healthcare. However, it is important to also address other financial issues like loss of income for the day it takes to visit the hospital and the fact that many might still experience financial hardship due to TB [31].

Surprisingly, only 4% of the participants knew that there is a vaccine for TB. The Bacille Calmette-Guerin (BCG) vaccine has a varying effect on adult pulmonary TB but is still widely used in children where it protects against disseminated disease [32]. According to WHO estimates, 89% of the Indian population was covered by the BCG vaccine in 2016 [33]. Most people are unaware of having had the vaccine perhaps because it is given in a very early stage of life [34].

### Characteristics and attitude

In general, the literacy rate among participants in this study was lower (61%) than the national average in India (74%) [35]. Additionally, the knowledge-score comparison establishes a correlation between education level and TB knowledge among the participants with a significantly higher mean score among those with at least ten years of schooling (25% of participants) compared with the illiterates. Our findings, therefore, suggest that the illiterate pilgrims attending the *Kumbh Mela* are significantly more vulnerable to the delays in TB diagnosis and initiating treatment due to their lower knowledge levels.

The composite results suggest some significant differences in attitudes between male and female participants. More females considered that it is not possible to live a normal life with TB and considered TB to be more dangerous than cancer compared with males (*p* > 0.05). Thus, one might conclude that males had a more optimistic view of the disease than females. However, more males than females had a problem living with a person with TB, suggesting a more discriminating view amongst males. In the larger study on stigmatizing attitudes on TB in India by Sagili et al., no gender difference in discriminatory views was reported [12]. Therefore, further research into gendered attitudes against TB-patients should be conducted before drawing too far-reaching conclusions.

All pilgrims participating in this study indicated that they planned to bathe in the holy river during the *Kumbh Mela*, indicating a strong adherence to the religious tradition. Interestingly, as many as 12% responded that bathing could potentially cure TB. This shows a still enduring belief in the power of this ritual. Additionally, even if many were unsure, only 1% thought that there was any risk of getting infected if they were bathing with a TB patient, hence depict underestimating the risks associated with the MG.

### Determinants of the knowledge-score

Education and occupation significantly contributed to an individuals’ knowledge-scores. Self-employment correlated with lower scores than the housewives, privately employed participants and students. Perhaps this also reflects an educational difference between groups, but since the different categories have not been further defined and data on household income was not provided, this is only speculation.

Interestingly, those who had travelled the furthest (from a state other than Madhya Pradesh) had significantly lower knowledge-scores than those living closer to the study setting. One could otherwise assume that ability to travel was a sign of higher socioeconomic status and hence higher education levels. However, this difference might be due to the previous awareness campaigns about the risk of transmitting communicable diseases held in the study area. Additionally, those with the highest scores were living in Ujjain city itself, potentially representing a more urbanized and educated group. There is a need for more focused research to define the factors affecting these results.

### Transmission of TB and the MG aspect

Even though the results suggest that most pilgrims are aware of the risk of TB-exposure when being in contact with a patient, only 4% regarded being in a crowded area as a potential risk factor for TB transmission. Attending an MG (*Hajj*) has been presented as an increased risk of getting infected by someone with active TB, as well as having a previously latent infection reactivated [18, 36]. For the *Kumbh Mela* 2016, a system for disease surveillance was implemented where 28% of total symptoms reported were of acute respiratory illness, suggesting a potential for droplet transmission at the event [25]. Hence, it would be fair to infer that many pilgrims underestimated the risk of attending such an event and perhaps might have failed to take necessary precautions. This was further confirmed by the low response rates for not spitting and covering one’s face while coughing as two of the ways to prevent the spread of TB. The issue of spitting, a common behavior in South Asia sometimes associated with chewing tobacco, has been brought to the fore recently as a potential route of Covid-19 transmission [37]. Intensified efforts to reduce open spitting among the population because of the current pandemic could also have a beneficial effect on TB-transmission. Increased knowledge could be an approach to increase acceptance for afflicted individuals. Thus, there is an absolute need for more research, reframing the policies and educational contents, using media and strengthening the health system to successfully tackle these challenges in the future.

#### Strengths and limitations

The primary strength of this study is the sample size. The participants had a variety of demographic characteristics, that provided an opportunity to reliably explore the TB-knowledge, discover statistically significant differences between for example males and females, and hence draw important conclusions from the results among this population. Another strength is the method used for data collection used in one of the world’s largest MG. The method can be easily reproduced at future MG using the same questionnaire. However, the use of a non-probabilistic sampling method is a limitation to this study design. An effort was made to amend this by diversifying data collectors, locations, and time-points, but the probability of adaptation of convenience sampling could not be denied. This could lead to potential selection bias as well as making inferential statistics less reliable. Besides rotational shift work and random selection method used to approach potential study participants, our results show low numbers of participants from the states other than Madya Pradesh, who probably attended the *Mela* but could not be fully reflected in the results. However, the special circumstances of the *Kumbh Mela*, with crowds of hundreds of people visiting and constantly on the move, makes other approaches to sampling very challenging. Moreover, the aim of the study was not to compare pilgrims’ knowledge based on their place of residence.

Another potential limitation is the use of locally developed questionnaires in this type of research. This limits the control for response biases by comparing with previous studies. However, most of the questions were responded hence, this project could set a standard for future investigations in this area enabling research on how TB-knowledge changes over time or between locations.

It is worth stating the social desirability bias which influences the responses in such studies, especially towards the attitude related questions, since the respondents generally want to appear sympathetic and correct. To overrule this, the participants were informed about anonymity during the introduction therefore, this was probably not a decisive issue.

There were very low response rates to some of the open questions. Therefore, they could not be included in the study. Perhaps this could be explained by time restraints on behalf of the participants, who were on their way to or from the river or other activities. Since this was anticipated, most questions had alternative answers (multiple choice questions) to choose from. To get fuller responses to the open questions the pilgrims could have been taken aside for a longer interview. However, this would have resulted in fewer participants in the study. It would be interesting to conduct a qualitative study at a future *Kumbh Mela* to further explore the reasoning by the pilgrims around infection risks and adherence to preventive measures.

#### Future implications

The results from this study could be used by the authorities and policymakers of the MG to make amendments for the weaker areas regarding TB. Since most pilgrims were aware of the disease and some of its most prominent characteristics, it would be fair to conclude that the previous information campaigns have been successful to a certain extent. However, it also becomes evident that some information, like knowledge of long-term symptoms, affordability of treatment and preventive behaviors, needs to be focused on further.

From the MG aspect, future research ought to investigate measurements of actual TB-burden MG such as the *Kumbh Melas,* to increase awareness and allow pilgrims to make informed decisions about their attendance. The effects of implemented policies are to be evaluated through follow-up research. Inspiration can be drawn from the plentiful studies of the *Hajj*, but methods need to be adapted to the special circumstances of the Indian settings. Screening programs for active TB would be desirable but perhaps still unaffordable and practically difficult to implement due to the enormous size of the crowds. Therefore, further increasing everyone’s knowledge and empowering them to take responsibility for their own and their fellow citizens’ health, might be the more plausible way to go.

## Conclusion

This study provides new insight into the knowledge of TB among the attendees of a religious MG in India. It shows that some basic characteristics of the disease were widely known in this population. However, some remaining knowledge-gaps were also detected, especially concerning long-term symptoms of TB and important preventive measures. Awareness of the risk of TB transmission associated with MG such as the *Kumbh Mela* was in general, assessed to be inadequate. To reach the WHO’s targets for ending the TB epidemic by 2030, more detailed knowledge about the disease and how to prevent its spread needs to be conveyed to the Indian population. Information campaigns should put extra effort into reaching illiterates and those with low levels of education, empowering them to seek care early in the disease progression and to avoid unnecessary contamination among their peers.

## Data Availability

All data generated or analyzed during this study are included in this published article. The datasets generated and/or analyzed during the current study are not publicly available as it contains personal information of the participants. However, the information was blinded before data analyses. The data are available from the corresponding author on reasonable request.

## Abbreviations

AIDS: Acquired Immunodeficiency Syndrome
ANOVA: Analysis of Variance
BCG: Bacille Calmette-Guerin
IEC: Institutional Ethics Committee
MG: Mass gatherings
NSP for TB elimination: National Strategic Plan for Tuberculosis Elimination
RDGMC: R.D. Gardi Medical College
RNTCP: Revised National Tuberculosis Control Program
TB: Tuberculosis
UNESCO: United Nations Educational, Scientific and Cultural Organization
WHO: World Health Organization

## References

[1] World Health Organization. End TB Strategy - Global strategy and targets for tuberculosis prevention, care and control after 2015. Geneva; 2014. Available from: https://www.who.int/tb/strategy/End_TB_Strategy.pdf.

[2] World Health Organization. Global Tuberculosis Report. Geneva. 2020:2020. Available from: https://www.who.int/publications/i/item/9789240013131.

[3] The World Bank. Country profile: India [Internet]. [cited 2021 Feb 3]. Available from: https://data.worldbank.org/country/india.

[4] National Strategic Plan for Tuberculosis Control 2012-2017. Ministry of Health and Family Welfare. New Delhi: Central TB Division, Directorate General of Health Services, Ministry of Health & Family Welfare; 2014. Available from: https://www.tbfacts.org/wp-content/uploads/2016/01/NSP-2012-2017.pdf.

[5] Storla DG, Yimer S, Bjune GA (2008). A systematic review of delay in the diagnosis and treatment of tuberculosis. BMC Public Health.

[6] Mbuthia GW, Olungah CO, Ondicho TG. Health-seeking pathway and factors leading to delays in tuberculosis diagnosis in west Pokot County, Kenya: A grounded theory study. PLoS One. 2018;13(11). 10.1371/journal.pone.0207995. 10.1371/journal.pone.0207995 PMC6261612 30485379

[7] Golub JE, Bur S, Cronin WA, Gange S, Baruch N, Comstock GW, Chaisson RE (2006). Delayed tuberculosis diagnosis and tuberculosis transmission. Int J Tuberc Lung Dis.

[8] Miller AC, Arakkal AT, Koeneman S, Cavanaugh JE, Gerke AK, Hornick DB, Polgreen Philip M . Incidence, duration and risk factors associated with delayed and missed diagnostic opportunities related to tuberculosis: a population-based longitudinal study. BMJ Open 2021;11(2):2. 10.1136/bmjopen-2020-045605. 10.1136/bmjopen-2020-045605 PMC7896623 33602715

[9] Huddart S, Bossuroy T, Pons V, Baral S, Pai M, Delavallade C (2018). Knowledge about tuberculosis and infection prevention behavior: a nine city longitudinal study from India. PLoS One.

[10] Westerlund EE, Tovar MA, Lönnermark E, Montoya R, Evans CA (2015). Tuberculosis-related knowledge is associated with patient outcomes in shantytown residents; results from a cohort study. Peru J Infect.

[11] Mbuthia GW, Olungah CO, Ondicho TG. Knowledge and perceptions of tuberculosis among patients in a pastoralist community in Kenya: a qualitative study. Pan Afr Med J. 2018;30. 10.11604/pamj.2018.30.287.14836. 10.11604/pamj.2018.30.287.14836 PMC6317384 30637071

[12] Sagili KD, Satyanarayana S, Chadha SS. Is knowledge regarding tuberculosis associated with Stigmatising and discriminating attitudes of general population towards tuberculosis patients? Findings from a community based survey in 30 districts of India. PLoS One. 2016;11(2). 10.1371/journal.pone.0147274. 10.1371/journal.pone.0147274 PMC4734597 26829713

[13] Public health for mass gatherings: key considerations [Internet]. Geneva; 2015 [cited 2021 Apr 2]. Available from: https://www.who.int/publications/i/item/public-health-for-mass-gatherings-key-considerations.

[14] Memish ZA, Alrabeeah AA (2011). Jeddah declaration on mass gatherings health. Lancet Infect Dis.

[15] Yezli S, Assiri A, Nabulsi H, Awam A, Blumberg L, Endericks T, Stergachis A, Reicher S, McCloskey B, Petersen E, Alotaibi B (2018). From mass gatherings medicine to mass gatherings health: conclusions from the 3rd international conference on mass gatherings medicine, Riyadh, Kingdom of Saudi Arabia.

[16] Petersen E, Memish ZA, Zumla A, Al MA (2020). Transmission of respiratory tract infections at mass gathering events. Curr Opin Pulm Med.

[17] Zumla A, Saeed A Bin, Alotaibi B, Yezli S, Dar O, Bieh K, Bates M, Tayeb T, Mwaba P, Shafi S, McCloskey B, Petersen E, Azhar EI. Tuberculosis and mass gatherings—opportunities for defining burden, transmission risk, and the optimal surveillance, prevention, and control measures at the annual hajj pilgrimage. Int J Infect Dis 2016;47(May 2014):86–91. 10.1016/j.ijid.2016.02.003. 10.1016/j.ijid.2016.02.003 26873277

[18] Wilder-Smith A, Foo W, Earnest A, Paton NI (2005). High risk of mycobacterium tuberculosis infection during the hajj pilgrimage. Trop Med Int Heal.

[19] Alqahtani AS, Alfelali M, Arbon P, Booy R, Rashid H. Burden of vaccine preventable diseases at large events. Vaccine. 2015;33(48):6552–63. 10.1016/j.vaccine.2015.09.076. 10.1016/j.vaccine.2015.09.076 26437018

[20] Memish ZA, Steffen R, White P, Dar O, Azhar EI, Sharma A, Zumla A (2019). Mass gatherings medicine: public health issues arising from mass gathering religious and sporting events. Lancet..

[21] Dwivedi S, Cariappa MP (2015). Mass-gathering events: the public health challenge of the Kumbh Mela 2013. Prehosp Disaster Med.

[22] Baranwal A, Anand A, Singh R, Deka M, Paul A, Borgohain S, Roy N. Managing the earth’s biggest mass gathering event and wash conditions: Maha kumbh mela (India). PLoS Curr. 2015;7(DISASTERS). 10.1371/currents.dis.e8b3053f40e774e7e3fdbe1bb50a130d PMC4404264 25932345

[23] Sridhar S, Gautret P, Brouqui P (2015). A comprehensive review of the Kumbh Mela: identifying risks for spread of infectious diseases. Clin Microbiol Infect.

[24] UNESCO. Kumbh Mela - Intangible heritage [Internet]. 2017 [cited 2020 Nov 21]. Available from: https://ich.unesco.org/en/RL/kumbh-mela-01258.

[25] Goel P, Dhuria M, Yadav R, Khasnobis P, Meena S, Venkatesh S (2020). Public health surveillance during Simhastha Kumbh, a religious mass gathering in Ujjain district, Madhya Pradesh, India, 2016. Indian J Public Health.

[26] David S, Roy N (2016). Public health perspectives from the biggest human mass gathering on earth: Kumbh Mela. India Int J Infect Dis.

[27] Sharma N, Basu S, Chopra KK (2019). Achieving TB elimination in India: the role of latent TB management. Indian J Tuberc.

[28] Home: Central TB Division [Internet]. Government of India. [cited 2021 Apr 5]. Available from: https://tbcindia.gov.in/index.php.

[29] Sreeramareddy CT, Harsha Kumar HN, Arokiasamy JT (2013). Prevalence of self-reported tuberculosis, knowledge about tuberculosis transmission and its determinants among adults in India: results from a nation-wide cross-sectional household survey. BMC Infect Dis.

[30] Friday essay: coughs on film and the fine but deadly art of foreshadowing [Internet]. The Conversation. [cited 2021 Apr 11]. Available from: https://theconversation.com/friday-essay-coughs-on-film-and-the-fine-but-deadly-art-of-foreshadowing-135697.

[31] Yadav J, John D, Menon G (2019). Out of pocket expenditure on tuberculosis in India: do households face hardship financing?. Indian J Tuberc.

[32] Fatima S, Kumari A, Das G, Dwivedi VP (2020). Tuberculosis vaccine: a journey from BCG to present. Life Sci.

[33] WHO-UNICEF estimates of BCG coverage [Internet]. World Health Organization. [cited 2021 Apr 5]. Available from: https://apps.who.int/immunization_monitoring/globalsummary/timeseries/tswucoveragebcg.html.

[34] Universal Immunization Programme [Internet]. National Health Mission. [cited 2021 Apr 11]. Available from: http://www.nrhmhp.gov.in/content/immunisation.

[35] Profile - Literacy - Know India: National Portal of India [Internet]. The indian Government. [cited 2021 Apr 5]. Available from: https://knowindia.gov.in/profile/literacy.php.

[36] Yezli S, Zumla A, Yassin Y, Al-Shangiti AM, Mohamed G, Turkistani AM, Alotaibi B (2017). Undiagnosed active pulmonary tuberculosis among pilgrims during the 2015 hajj mass gathering: a prospective cross-sectional study. Am J Trop Med Hyg.

[37] Kar SK, Pandey P, Singh N (2020). Understanding the psychological underpinning of spitting: relevance in the context of COVID-19. Indian J Psychol Med.

